# *In Vitro* Apoptotic Effects of Farnesyltransferase blockade in Acute Myeloid Leukemia Cells

**Published:** 2016-11-01

**Authors:** V Giudice, P Ricci, L Marino, M Rocco, G Villani, M Langella, L Manente, E Seneca, I Ferrara, L Pezzullo, B Serio, C Selleri

**Affiliations:** 1 Department of Medicine and Surgery, University of Salerno, Baronissi, Italy; 2 Department of Clinical Medicine and Surgery, Federico II University of Naples, Naples, Italy

**Keywords:** acute myeloid leukemia, farnesyltransferase inhibitors, apoptosis, nitric oxide

## Abstract

Farnesyltransferase inhibitors (FTIs) are a class of oral anti-cancer drugs currently tested in phase I-II clinical trials for treatment of hematological malignancies. The *in vitro* effects of various FTIs (alpha-hydroxyfarnesylphosphonic acid, manumycin-A and SCH66336) were tested on CD34^+^ KG1a cell line and in primary acute myeloid leukemia (AML) cells from 64 patients. By cell viability and clonogeneic methylcellulose assays, FTIs showed a significant inhibitory activity in CD34^+^ KG1a and primary bone marrow (BM) leukemic cells from 56% of AML patients. FTIs also induced activation of caspase-3 and Fas-independent apoptosis, confirmed by the finding that inhibition of caspase-8 was not associated with the rescue of FTI-treated cells. We concluded that other cellular events induced by FTIs may trigger activation of caspase-3 and subsequent apoptosis, but the expression of proapoptotic molecules, as Bcl-2 and Bcl-XL, and antiapoptotic, as Bcl-X(s), were not modified by FTIs. By contrast, expression of inducible nitric oxide synthase (iNOS) was increased in FTI-treated AML cells. Our results suggest a very complex mechanism of action of FTIs that require more studies for a better clinical use of the drugs alone or in combination in the treatment of hematological malignancies.

## I. INTRODUCTION

In recent years, several target therapies have revolutionized the approach to various hematological malignancies [1–3]. Farnesyltransferase inhibitors (FTIs), a class of oral active anti-cancer drugs, can competitively inhibit the farnesyltransferase (FTase) enzyme [4–9]. FTIs have been tested in several myeloid hematological diseases, as acute and chronic myeloid leukemia (AML, CML), because of their overexpression of Ras, MAPK, AKT and other FTase-dependent proteins involved in proliferation, survival and apoptosis [10–21]. The Ras proteins mediate signals from multiple cell surface receptors to the nucleus via different effector cascades [16–21]. Cytoplasmic Ras proteins need to be prenylated for fully biological functions. Farnesylation by the housekeeping enzyme FTase is the first required step in *ras* prenylation, enabling membrane anchor, *ras* activation and Ras-mediated activation of its downstream effectors [7–8,20–23]. FTase enzyme transfers a lipophilic farnesyl group from farnesyl diphosphate (FDP) to thiol group of cystein terminal portion of Ras, known as “CAAX-tetrapeptide motif”, where “C” is cysteine, “A” is typically an aliphatic amino acid, and “X” is a serine or methionine as substrates of FTase. The other two additional steps of Ras prenylation are mediated by geranylgeranyl-transferase type I and type II enzymes (GGTase I and II) [7–8,19–23]. As other G proteins, also Ras activation is mediated by membrane guanine nucleotide exchange factors (GNEF), which allow Ras to cycle between an inactive guanosine diphosphate(GDP)- to an active guanosine triphosphate (GTP)-binding status [24]. Besides, the intrinsic GTPase activity of Ras can revert the molecule back to its GDP-inactive form. Mutations in *N-* and *K-ras* genes, documented in 15–30% of AML patients, can constitutively active Ras, which appears to be also involved in leukemogenic transformation by *bcr/abl* in CML [25–29].

In the last years, many FTIs have been identified, grouped into three main classes [30–31], and several of these have been evaluated preclinically. FDP analogues, as alpha-hydroxyfarnesyl phosphonic acid, beta-hydroxyphosphonic acid derivatives, PD 169451 or RPR 130401, can compete with FDP for the binding to FTase, but no specific antitumor effects have been described *in vivo*. CAAX competitive inhibitors, as L-778123, SCH66336 and R115777, are effective and selective FTase inhibitors *in vitro* and are currently testing *in vivo*. Bisubstrate inhibitors, as BMS 186511 and BMS-214662, combine the structural motifs of FPP and the CAAX tetrapeptide, linked by a phosphinyl or phosphonyl binding [30–31]. In particular, SCH66336 and R115777 oral compounds are testing in phase I and II clinical trials in haematological malignancies, including AML, CML, juvenile myelomonocytic leukemia (JMML), myelodysplastic syndromes (MDS) and multiple myeloma (MM), myelofibrosis with myeloid metaplasia and elderly high-risk AML [25–29,32–38]. These preclinical trials have been shown a Ras-independent FTI activity [30–31,39], probably because inhibition of Ras isoprenylation may be not necessary for FTI antineoplastic effects [40–41].

The *in vitro* cytotoxic effects of various FTIs have been previously described, including the NO-mediated apoptotic effects of SCH66336 in CML cells [42–43], but FTIs alone have shown only limited activity in AML and MDS patients [44–46]. The aim of the present study was to clarify the mechanism of action of FTIs, in order to explain the not enthusiastic results obtained in *in vivo* experiments. Consequently, we studied the *in vitro* effects of FTIs on apoptosis and growth of AML primary cells by blocking the downstream and upstream proteins of Ras-mediated apoptosis pathways.

## II. METHODOLOGY

### Compounds blocking farnesyltransferase

FTIs tested were: a nonpeptidic tryciclic competitive FTI SCH66336 (SCH) (a gift from Schering-Plough Research Institute, Kenilworth, NJ); alpha-hydroxy-farnesylphosphonic acid (α-HFPA) and manumycin-A (Man-A) (Sigma-Aldrich, St Louis, MO). These FTI compounds were stored at −20°C as 10 mM stocks in dimethyl sulfoxide (DMSO, Sigma-Aldrich).

### Cell lines and AML patient samples collection

KG1a cells (American Type Culture Collection, Manassas, VA), were grown in RPMI 1640 supplemented with 10% heat-inactivated fetal calf serum (FCS), 2 mM L-glutamine, 100 U/mL penicillin, and 0.1 mg/mL streptomycin (complete medium, CM) at 37°C in 5% CO_2_. Heparinized BM samples were obtained from 11 normal BM transplantation donors and during diagnostic procedures from 64 patients (median age, 52 years; range, 19–82; male/female, 28/36) with de novo (n=38) AML and AML with MDS-related features (n=26). Before sampling, informed consent was obtained according to the Declaration of Helsinki [47] and the procedures outlined by the ethical committee of our institution. Bone marrow mononuclear cells (BMMNCs) were isolated by density gradient centrifugation. After washing with phosphate-buffered saline (PBS; Life Technologies, Carlsbad, USA), BMMNCs were resuspended in Iscove’s modified Dulbecco’s Medium (IMDM) supplemented with 10% heat-inactivated FCS. RPMI 1640, IMDM, PBS, FCS, L-glutamine, penicillin, streptomycin, and lymphocyte separation medium were purchased from Life Technologies (Gaithersburg, MD).

### Suspension cultures

KG1a and primary AML cells were placed in 24-well plates in RPMI containing 0.1% FCS for 12 and 2 hours, respectively, before exposure to FTIs. For functional experiments, AML cells were preincubated for 2 hours with the following reagents: caspase-3 inhibitor Z-DEVD-FMK, caspase-8 inhibitor IETD-FMK, (all used at 50 μM and purchased from Alexis, San Diego, CA), Fas-receptor triggering inhibitor Fas:Fc (50 μM) (Alexis), iNOS inhibitor NG-monomethyl-arginine (500 μM) (γ-MM-arg; Calbiochem, San Diego, CA). All experiments were repeated at least 3 times and each experimental condition was repeated at least in duplicate wells in each experiment. All the incubations were conducted at 37°C with 5% CO_2_.

### Proliferation assay

*In vitro* sensitivity of KG1a and primary BM AML cells to FTIs was determined by plating 5 × 10^5^ cells in RPMI 0.1% FCS and several dilutions of FTIs in 24-well plates. Controls were performed using identical concentrations of the solvent used for FTIs. After 48h incubation, cell viability was determined by MTS (3-(4,5-dimethylthiazole-2yl)- 5- (3-carboxymethoxyphenyl)- 2- (4-sulfophenyl)- 2H-tetrazolium, inner salt) and PES (phenazine ethosulfate) assay (CellTiter 96 AQ_ueous_ One Solution Reagent) provided by Promega (Madison, WI, USA). Briefly, 20 μl of CellTiter 96 AQ_ueous_ One Solution Reagent was added to each well and incubated for 4 hours. The optical density (OD) was measured at 490 nm using a microplate spectrophotometer (Titertek Multiscan, BioRad, Hercules, CA). FTI effects were measured as percentage of inhibition of cell viability by the following equation: 1−[(OD treated well/mean OD control wells) × 100], after background correction using blank wells. Each experiment was performed in triplicate. Values were expressed as mean value ± standard error of mean (SEM). The results were considered evaluable only if the control wells contained at least 70% of live AML cells after 48 h culture.

### Hematopoietic colony assay

Short-term clonogenic progenitors were measured in methylcellulose (Stem Cell Technologies, Vancouver, CA). Isolated BMMNCs were plated in methylcellulose at 1 × 10^3^ cells/ml concentration (35 mm dishes; 1 ml of medium/dish) in the presence of the following cocktail: 10 ng/mL IL-3, 50 ng/mL G-CSF, 50 ng/mL GM-CSF, 20 ng/mL SCF, and 2 U/mL EPO. Anti-Fas antibody (clone CH11, Amac) was used at concentrations ranging from 20–1000 U/ml to 1 μg/ml. Myeloid and erythroid colonies and clonogeneic aggregates of less than 50 cells, were counted as granulocyte-macrophage colony forming unit (CFU-GM), erythroid-burst forming unit (BFU-E) and clusters, respectively, after 14-day incubation. Each experiment was done in quadruplicate. Values were expressed as mean value ± standard error of mean (SEM).

### Apoptosis assays

DNA fragmentation was measured after low molecular weight (LMW) DNA extraction from 2 × 10^6^ cells as previously described [48]. The high molecular weight DNA fraction was precipitated for 6 h in the presence of 5 mol/L NaCl and pelletted by high-speed centrifugation. The fragmented DNA was extracted, precipitated and electrophoresed as previously described [48].

Flow cytometric analysis of cellular DNA was performed using propidium iodide (PI) staining as previously described [48], adding 100 U/mL RNAse A (Boehringer Mannheim, Mannheim, Germany) during the incubation step. A minimum of 60,000 events was counted per sample. Apoptotic cell nuclei containing hypodiploid (fragmented) DNA were counted as a percentage of total population.

### Fas-R/FasL flow cytometry analysis

Phycoerythrin (PE)-conjugated - CD34 (clone HPCA-1; Becton Dickinson [BD], San Jose, CA) was used to identify CD34^+^ cells. A fluorescein isothiocyanate (FITC)-conjugated - fragment of a murine anti-human CD95 (clone UB2; Amac, Westbrook, ME) combined with a PE-conjugated - CD34 was used to determine the expression of Fas-R on CD34^+^ cells. For Fas-L expression, BM cells were incubated with metalloproteinase inhibitor KB8301 (10 μmol/L, Pharmingen, San Diego, CA), PE-conjugated - CD34 (BD) combined with a purified mouse anti-human Fas**-**L (IgG1, Nok1; Pharmingen) for 1 hour at 4°C. Then cells were incubated with FITC-conjugated goat anti-mouse IgG secondary antibody for 30 minutes at 4°C. Proper isotypic controls were used in all experiments.

### Detection of caspases 3 and 8 activities

Activation of intracellular caspases was performed by flow cytometry using fluorogenic caspase-specific substrates (DEVD-AFC for caspase-3, Alexis; and IETD-AFC for caspase-8, Pharmingen). Cells (1 × 10^6^) were treated with FTIs in the presence or absence of caspase-specific inhibitors for 24 hours, washed with PBS, and then resuspended in 50 μL of substrate buffer containing 10 mmol/L dithiothreitol (DTT) and 10 μL of the fluorogenic caspase-specific substrate supplemented with 5 μL of FCS. After centrifugation at 800g for 5 minutes, cells were incubated at 37°C for 60 minutes. After incubation, cells were washed and acquired. At least 10,000 events were analysed. Results were measured as fold increase in fluorescence relative to untreated control cells.

### Reverse transcription-polymerase chain reaction (RT-PCR) for human iNOS RNA

Total RNA was extracted from BMMNCs in TRIzol (Invitrogen, Carlsbad, CA), as previously described [48]. Human iNOS cDNA, after reverse transcription using an oligo d(T)16 primer, was amplified using the following primer pairs: 5′ CGGTGCTGTATTTCCTTACGAGGCGAAGAAGG-3′ and 5′-GGTGCTGCTTGTTAGGAGGTCAAGTAAAGGGC-3′. Human glyceraldehyde-3-phosphate dehydrogenase (GAPDH, 5′-TTCACCACCATGGAGAAGGCT-3′ and 5′-ACAGCCTTGGCAGCACCAGT-3′) was used as housekeeping gene. Reagents supplied in the Amplimer kit (Perkin Elmer, Foster City, CA) were used for the amplification reaction. PCR products were electrophoresed in 1.2% agarose gels and the bands were visualized after staining with ethidium bromide and UV-light exposure.

### Immunoblotting of iNOS, Bcl2, BclXL and p53

Treated AML cells were washed and lysed as previously described [48]. Concentrations were measured by colorimetric method (Biorad, Richmond, CO). A total of 100 μg of cell lysate, together with molecular weight markers (Amersham, Little Chalfont, UK) and iNOS positive mouse macrophages lysate (Transduction Laboratories, Lexington, KY) were fractionated by 7.5% SDS-polyacrylamide gel electrophoresis. PVDF membranes were prepared as previously described [48] and incubated with 2 μg/ml of mouse anti-iNOS, anti-Bcl2, anti-p53, anti-BclXL and anti-BclXS antibodies (Transduction Laboratories, Lexington, KY) overnight at 4°C. The reaction was revealed by incubating filters with horseradish peroxidase-conjugated goat anti-rabbit antibodies (BioRad, Richmond, CO) and developed by ECL (Amersham, Little Chalfont, UK) according to manufacturer’s specifications.

### Statistical analysis

Two-tailed Student t-test was used for flow cytometric analysis and tissue culture experiments. p values ≤ 0.05 were considered statistically significant.

## III. RESULTS

### FTI-induced growth inhibition of AML cells is partly related to apoptosis

We first investigated the effects of FTIs on growth of KG1a AML cell line. In cell viability tests performed in logarithmic growth phase, KG1a cell survival was inhibited by all three FTIs in a dose dependent manner. By linear regression analysis, we determined the 50% inhibitory concentration (IC50) after 48 hour exposure to SCH, Man-A and α-HFPA. On an equimolar concentrations, SCH displayed more toxicity than Man-A (IC50 mean, 5 μM *vs* 50 μM, respectively; range, 1–20 μM *vs* 25–150 μM, respectively) and α-HFPA (IC50 mean, 100 μM; range, 75–150). The observed mean IC50 concentrations were used for next experiments.

**Figure 1:**
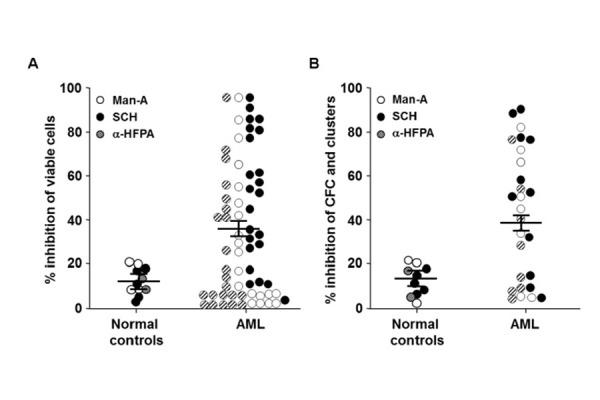
FTI-induced inhibition of AML cell viability and clonal growth. Viability and cluster/colonies of AML cells were detected by MTT (A) and methylcellulose assays (B) measured by colony forming cell (CFC) assays. Each dot (subject studied) represents the percentage of inhibition of cell viability and colony formation by each FTI at the IC50 described in the text (control cultures were considered 100%). Horizontal bars are the mean values, and vertical bars are SEMs. Cumulative mean inhibition of cell viability and CFC ± SEM, as well as statistical analysis, are reported in the corresponding results section. Abbreviations, see text.

The inhibitory effects of SCH, Man-A and α-HFPA were significantly stronger in primary AML cells than normal marrow cells (mean inhibition % ± SEM, 35±6 *vs* 13±5, respectively; p=0.03). We documented a FTI-mediated inhibition of cell viability >25% in 56% of AML patients (Fig. 1A). Similarly, by methylcellulose clonogeneic assays, colony and cluster growth inhibition >25% was observed in 64% of AML patients, whereas colony cells from normal BM cells were only weakly affected by FTIs exposure (mean inhibition % ± SEM, 47.7±7 *vs* 15.5±1, respectively; p<0.001) (Fig. 1B). By allele-specific PCR, the presence of oncogenic *N-Ras* and *K-Ras* mutations were also investigated in primary cells from 30 AML patients. Oncogenic mutations of *N-Ras* were detected in 27% of AML subjects; by contrast, we did not observe *K-Ras* mutations in any AML cell sample. Indeed, FTI-mediated inhibition of cell growth was observed in both AML with and without *N-Ras* mutations (data not shown).

**Figure 2:**
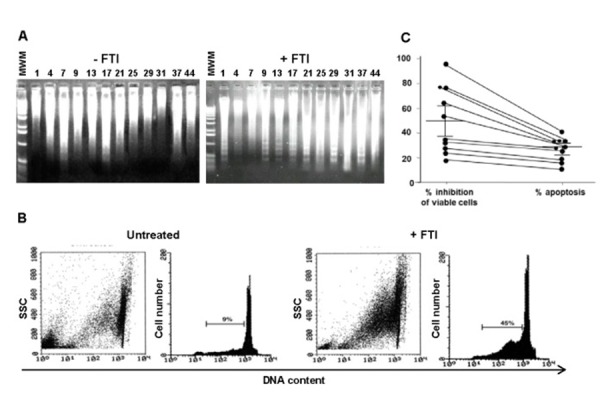
FTI-induced inhibition of AML cell proliferation is partly related to apoptosis. Agarose gel stained with ethidium bromide after electrophoresis of low molecular weight DNA from 12 representative BM AML patients exposed for 48 h to FTIs at the IC50 (A). A representative flow cytometric detection of apoptic hypodiploid DNA peak stained with PI from BM AML patient exposed to SCH (B). Percentages of viable cells and apoptotic cells simultaneously measured from 10 representative BM AML patients after exposure for 48 h to FTIs (C). Cumulative percentage of mean inhibition of cell viability and apoptotic cells, as well as statistical analysis, are reported in the corresponding results section. Abbreviations, see text.

To define whether the FTI-mediated growth inhibition of primary AML cells was associated to apoptosis, BM AML cells sensitive to FTI inhibition were exposed for 48 h to FTIs at IC50. By LMW DNA fragmentation analysis, AML cells showed the characteristic DNA ladder suggestive of apoptosis (Fig. 2A). Flow cytometric detection of apoptotic hypodiploid DNA peak derived from treated BM AML cells confirmed the FTI-enhanced apoptotic cytotoxic effect (Fig. 2B). Indeed, percentages of apoptotic AML cells were significantly lower than those with cytolysis measured by cell viability assay (mean % ± SEM of cytolysis, 55.7±10; p=0.03). The parallel measurement of the number of apoptotic AML cells and viable cells from 10 AML patients carried by flow cytometry and cell viability assays showed that the percentages of apoptotic AML cells were significantly lower than those undergoing cytolysis (mean % ± SEM of apoptotic and viable AML cells, 32 ± 7 *vs* 51 ± 11, respectively; p = 0.03) (Fig. 2C).

### Caspase-3 and Fas-independent pathways are involved in FTI-mediated apoptosis of AML cells

**Figure 3:**
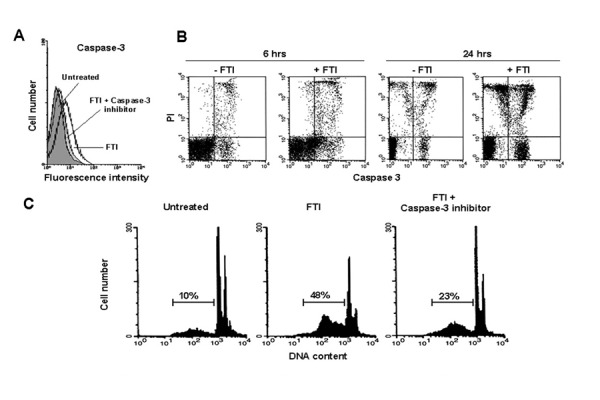
FTI-induced apoptosis in AML cells is mediated by activation of caspase-3. FTI induced activation of caspase-3. Caspase-3 activity in BM AML cells from a representative patient, cultured for 24 h in medium alone (untreated) or treated with FTI or with FTI + caspase-3 inhibitor Z-DEVD-FMK (50 μM), were measured by flow cytometry using the fluorogenic caspase-3 specific substrate DEVD-AMC (A). Caspase-3 is activated after FTI exposure in AML cells. Caspase-3 production in BM AML cells from a representative case, cultured for 6 and 24h in control medium (−FTI) or treated with FTI (+ FTI), was measured by flow cytometry using the cell-permeable FITC-labeled peptide FAM-VAD-FMK in combination with PI to differentiate dead from live cells (B). After 6 and 24 h of FTI exposure, 33% and 55% of live AML cells (B, bottom right panels) are positive for FAM-VAD-FMK, respectively. (Panel C) FTI-mediated apoptosis of AML cells is partially reverted by caspase-3 inhibition. Flow cytometric detection of apoptosis from a representative AML patient who showed in vitro susceptibility to FTI (SCH 1 μM) (C). Abbreviations, see text.

FTIs could induce caspase-3 activation by flow cytometric measurement of MFI after cleavage of the specific fluorogenic substrate DEVD-AFC (mean percentage of positive cells after 6 and 24h culture, 25±4% and 38±4% *vs* 9±1% and 18±5%, FTI-treated and control AML cells, respectively; mean of 5 experiments). In addition, pre-incubation with caspase-3 inhibitor Z-DEVD-FMK allowed to partially abrogate FTI-mediated caspase activation (mean % ± SEM after 24 h exposure to FTI, 22±6; p=0.03) (Fig. 3A–B), and also to partially prevent FTI-mediated apoptosis (mean % of apoptosis ± SEM after FTI treatment, 36±4 *vs* 16.3±5, in absence and in presence of Z-DEVD-FMK respectively; p=0.01) (Fig. 3C).

**Figure 4:**
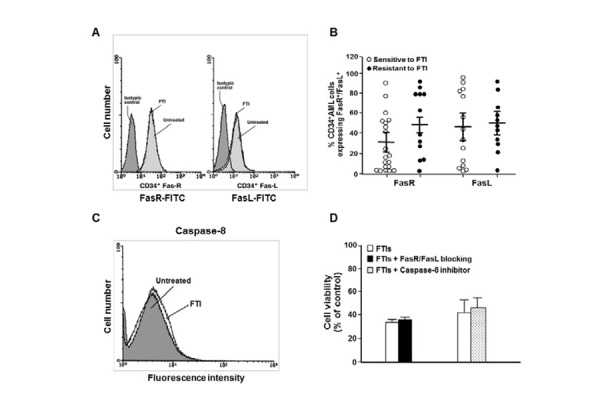
FasR/FasL pathway is not involved in FTI-induced apoptosis in AML cells. Flow cytometric analysis of FasR and FasL expression in CD34^+^ AML cells from a representative case, untreated and treated with FTI (A). FTI-mediated inhibition of AML cell viability does not correlate with FasR and FasL expression on CD34+ AML cells. Cumulative mean ± SEM CD34+ Fas-R+ and C34+ Fas-L+ are reported (B). Caspase–8 is not activated by FTIs. AML cells untreated and treated with FTI after 48 h of culture. (C). AML cells were grown for 48 h with FTIs at IC50 and with FTIs + Fas-receptor triggering inhibitor Fas:Fc or caspase-8 inhibitor IETD-FMK (all used at 50 μM). AML cell viability was measured by colorimetric MTT assay. Columns represent cumulative mean ± SEM of cell viability of 5 experiments performed with all FTIs (D).

Fas-R and Fas-L expression on AML cells exposed to FTIs was analysed by flow cytometry, but no variations were observed (21±4% and 23±8% in the absence and presence of FTIs, respectively) (Fig. 4A). Moreover, FTI–sensitive and FTI-resistant AML cells displayed similar expression of Fas-R (mean % ± SEM, 39±4 *vs* 49±6, respectively; p=0.5) and Fas-L (mean % ± SEM, 49±4 *vs* 52±3, respectively; p=0.7) (Fig. 4B). No modifications of caspase-8 activity were detected in AML cells after 48 h FTIs exposure by flow cytometry and intracellular caspase staining (Fig. 4C). To further exclude the involvement of Fas-R/Fas-L system in FTI-mediated apoptosis, prior to exposure to FTIs, AML cells were preincubated for 2 h with selective Fas-R/Fas-L inhibitors. As expected, no variations were observed in FTI-mediated inhibition of cell growth and apoptosis after pretreatment with Fas-receptor antagonist and caspase-8 inhibitor (mean % ± SEM of cell growth after FTI exposure, 41.6±3 and 43.3±3 without and with Fas:Fc *vs* 38±2 and 39±1 without and with IETD-FMK *vs* 42.3±3 and 46.6±3 without and with ZYVAD-FMK, respectively) (Fig. 4D).

### FTIs trigger iNOS expression in AML cells

**Figure 5:**
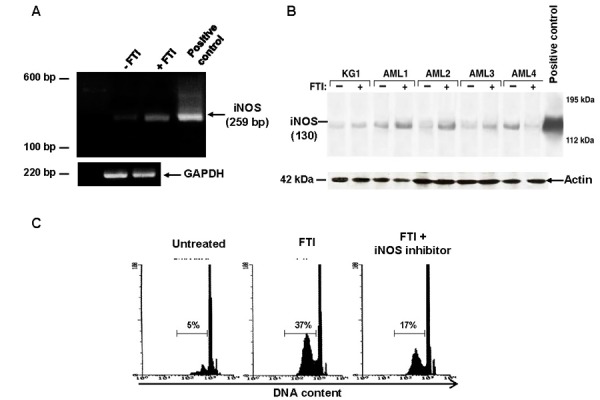
FTI-induced inhibition of CML cell viability is mediated by NO pathway. FTIs induce iNOS mRNA signal amplification in KG1a and AML cells cultured for 48h. Ethidium bromide RT-PCR products (iNOS and GAPDH) are shown (A). FTIs cause increased expression of iNOS protein in KG1a cell line and 4 AML patients. Actin is used as control (B). γ-MM-arg partially preventes FTI-mediated apoptosis. Flow cytometric detection of apoptic hypodiploid DNA peak stained with PI from a CML patient treated with FTI (C).

Unstimulated primary total AML BM cells expressed iNOS mRNA by PCR detection. Because iNOS expression in total BM AML cells might be related to non-leukemic cells, immature CD34^+^ KG1a cells were tested for the expression of iNOS mRNA after 48 h culture. A stronger amplification of iNOS was detected, and also after FTIs stimulation (Fig. 5A). Similarly, immunoblot of cell lysates showed lower levels of iNOS at baseline, and a 10-fold increased after FTIs stimulation in both primary total BM AML cells and KG1a cells (Fig. 5B). Using the cell-permeable fluorescent indicator DAF-2 DA, NO levels increased 40% after FTI exposure from basal levels detected in AML BM cells cultured in control medium without FTI (p<0.001), and γ-MM-arg partially blocked FTI-mediated NO production in CML cells (Fig 5C). Inhibition of NO synthesis by pretreatment of AML cells with γ-MM-arg (500 μM), a competitive inhibitor of iNOS, did not affect the inhibitory effect on cell growth and apoptosis of FTIs (data not shown). However, when FTIs were used at IC10, γ-MM-arg partially prevented FTI effects (mean % ± SEM cell growth and apoptosis after FTI exposure, 31±2 and 21±4 *vs* 46±1 and 31±3 in absence and in presence of γ-MM-arg, respectively; p=0.01 and p=0.03) (Fig. 5C).

### FTI exposure may modify p53 but not Bcl-2 pathway in AML cells

**Figure 6:**
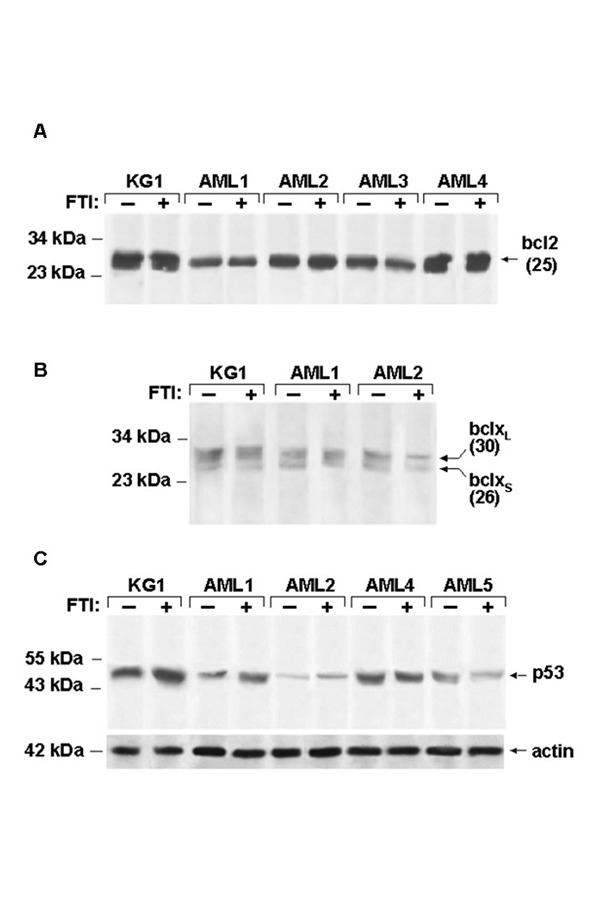
FTI exposure may modify p53 but not Bcl-2 pathway in AML cells Immunoblotting of Bcl2, BclXL and BclXs from KG1a cells and total marrow cells of four AML patients cultured for 48 h in absence or in presence of FTIs, excludes the involvement of Bcl-2 pathway in FTI-induced apoptosis in human AML cells (A-B). Immunoblotting of p53 from KG1a cells and total marrow cells of four AML patients cultured for 48 h in absence or in presence of FTIs, shows the enhancement of p53, as quantified by densitometry scanning, in 2 out of 4 AML cases after FTI exposure (C).

To explore whether FTI-induced apoptosis could be mediated by decreased expression of anti-apoptotic Bcl-2 and Bcl-X(L) or increasing in proapoptotic Bcl-X(S) proteins, AML cells were exposed for 48 hours to FTIs or control medium and Bcl-2 or Bcl-X(L)/(S) were measured. Quantification of protein banding by densitometry did not document changes in Bcl-2 protein expression in KG1a as well as in four primary BM AML samples after FTI exposure as compared to control cultures (Fig. 6A). Similarly, the expression of Bcl-X(L) and Bcl-X(S) did not show variations after FTI exposure (Fig. 5B). By contrast, in KG1a cells and few AML cases, an enhancement of p53 expression after FTI exposure was detected (Fig. 6C).

## IV. DISCUSSION

**Figure 7:**
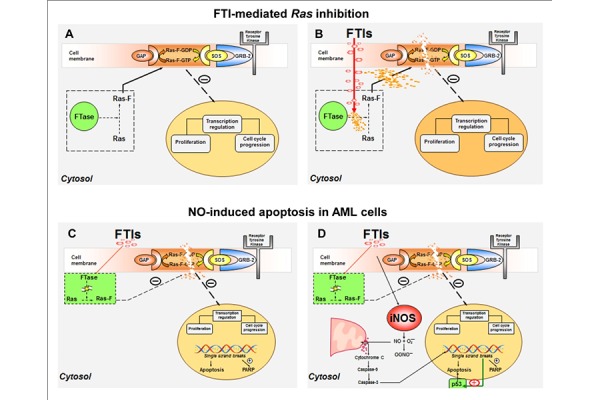
Models for FTI-mediated Ras inhibition (A–B) and NO-induced apoptosis (C–D) in AML. Detailed explanation is reported in Discussion. Abbreviations, see text.

Despite the improvements in achieving prolonged remission for adult AML after cytotoxic induction chemotherapy or BM transplantation, the overall survival remains unsatisfactory, particularly for older patients [1–3]. For this reason, new treatment approaches are required to improve the outcome in these patients. The upregulation of the classic mitogenic Ras/Raf/MAPK cascade has been documented in more than 30% of AML cases, due to mutations, overexpression or amplification of other oncogenic proteins with tyrosine kinase activity that may influence Ras pathways [10–21]. When farnesylation of Ras is blocked by FTIs *in vitro*, Ras is unable to anchor to cell membrane and its function is impaired (Fig. 7A–B) [16–21]. Various FTIs have already been evaluated in phase I-II and III clinical trials for the treatment of hematological malignancies, but contrasting results were reported, even though when associated to cytotoxic agents [25–29,32–38].

Here, the *in vitro* effects of FTIs were studied in AML cells. We have shown that FTIs showed a significant inhibitory activity on cell viability in CD34^+^ KG1a cell line and primary BM cells from 56% of AML patients. Indeed, only half of our AML patients showed increased sensitivity of cluster and colony growth after FTI exposure compared to normal marrow progenitor cells. Furthermore, the lack of response in the remaining half of AML patients could be related to the presence of mutations in FTase or other markers of resistance to FTIs, as p53 mutations, but more studies are needed to confirm this hypothesis [49–51].

Preliminary data have excluded that FTI-mediated inhibition of cell growth was *Ras*-mutations dependent, as FTIs induced inhibition of viability in AML cells with and without *N-Ras* mutations (Selleri et al., personal communication). As previously reported for CML cells, FTI-mediated cytotoxic effects in AML cells were partially related to enhanced apoptosis [48]. Although it has been reported that Ras-transformed cells showed Fas-R upregulation and higher Fas-related apoptosis after FTI-exposure [52–53], we documented a Fas-independent FTI-mediated apoptosis in AML cells. Moreover, FTI-mediated apoptosis seemed to be caspase-3 dependent but caspase-8 independent as inhibition of caspase-8 was not associated with the rescue of FTI-treated cells. These findings suggest that other cellular events induced by FTIs may trigger caspase-3 activation and subsequent apoptosis in AML cells. As the main event for caspase-3 activation is the release of cytochrome c from mitochondria, the expression of potential molecules modulating apoptosis via mitochondrial pathways were studied [54–55]. Expression of proapoptotic Bcl-2 and Bcl-X(L) and antiapoptotic Bcl-X(S) proteins were not modified by FTIs, except for the involvement of Bcl-2 pathway in FTI-induced apoptosis in human AML cells.

Another known mechanism that can concur in the inhibition of tumor growth is the macrophage-mediated NO release in the site of inflammation or in tumor environment, causing mitochondrial release of cytochrome c and apoptosis. In addition, it has been described that Ras inhibitors can increase NO-induced cell death [56–62]. In a similar manner, FTIs can induce apoptosis in CML cells, through cytochrome c release and caspase-3 activation, as previously documented [59,62]. As expected, FTIs also can induce iNOS expression in AML cells. Moreover, inhibition of NO synthesis partially abrogated the effects of FTIs on apoptosis suggesting that iNOS cascade may be only one of the possible mechanisms of FTI-mediated apoptosis in AML cells (Fig. 7 C–D). The enhancement of p53 expression in KG1a cell line and some AML cases suggested a complex mechanism of action of FTIs. Indeed, Moasser et al. has already reported a more susceptibility to FTIs in p53 wild-type breast cancer cell lines [63].

Because caspases, iNOS and p53 play an important role in several intracellular pathways and can be triggered by several different extracellular signals, it is hard to clearly understand the FTIs mechanisms, also due to their aspecific action [54–62]. Several pathways are deregulated in cancers, as JAK/STATs or Syk/Btk, allowing proliferation and survival of tumor cells [64–67]. Indeed, it has been reported that over-activity of growth factor signaling pathways in breast cancer did not correlate with sensitivity to FTIs [63]. On the other hand, FTIs have shown contradictory results in MDS an AML patients, particularly in elderly and poor-risk AML [68–72], but synergized actions were reported when FTIs were combined to tyrosine kinase inhibitors (TKIs) or other drugs, as simvastatin [73–77]. These findings suggest that FTIs can induce growth inhibition not only through apoptosis, but also interfering with proliferation pathways, enhanced when TKIs are used. Besides, the efficacy of ruxolitinib, a JAK1/2 inhibitor, or ibrutinib, a Btk inhibitor, has been described in several hematological malignancies, and more studies are ongoing [78–79]. For these reasons, to improve the therapeutic potential of FTIs alone or in combination with standard chemotherapy or new targeting therapies, it is important to better understand their mechanisms of action, as to investigate how they could impact on other pathways or vice versa.

## V. CONCLUSION

As already reported, *in vitro* FTIs effects on cell viability and apoptosis are caspase-3 and iNOS dependent in AML cells, and Fas-R/Fas-L or Bcl2 independent. But the involvement of p53 suggests a more complex mechanism in the susceptibility of FTI-mediated apoptosis in AML cells. It is also possible that FTIs may have only additive anti-neoplastic effects, requiring the combination with other anti-cancer agents to be effective in AML therapy. Our data open new questions about FTIs mechanism of action and their use in myeloid malignancies as single agents or in combination, in order to improve the outcome of hematological patients who cannot be treated with more aggressive treatments.
